# Indices of Paraoxonase and Oxidative Status Do Not Enhance the Prediction of Subclinical Cardiovascular Disease in Mixed-Ancestry South Africans

**DOI:** 10.1155/2014/135650

**Published:** 2014-03-27

**Authors:** M. Macharia, A. P. Kengne, D. M. Blackhurst, R. T. Erasmus, M. Hoffmann, T. E. Matsha

**Affiliations:** ^1^Division of Chemical Pathology, Faculty of Health Sciences, National Health Laboratory Service (NHLS) and University of Stellenbosch, Cape Town, South Africa; ^2^NCRP for Cardiovascular and Metabolic Diseases, South African Medical Research Council & and University of Cape Town, Cape Town, South Africa; ^3^Division of Chemical Pathology, University of Cape Town, Cape Town, South Africa; ^4^Department of Biomedical Sciences, Faculty of Health and Wellness Science, Cape Peninsula University of Technology, P.O. Box 1906, Bellville, Cape Town 7530, South Africa

## Abstract

We evaluated the association of indices of paraoxonase (PON1) and oxidative status with subclinical cardiovascular disease (CVD) in mixed-ancestry South Africans. Participants were 491 adults (126 men) who were stratified by diabetes status and body mass index (BMI). Carotid intima-media thickness (CIMT) was used as a measure of subclinical CVD. Indices of PON1 and oxidative status were determined by measuring levels and activities (paraoxonase and arylesterase) of PON1, antioxidant activity (ferric reducing antioxidant power and trolox equivalent antioxidant capacity), and lipid peroxidation markers (malondialdehyde and oxidized LDL). Diabetic subjects (28.9%) displayed a significant decrease in PON1 status and antioxidant activity as well as increase in oxidized LDL and malondialdehyde. A similar profile was apparent across increasing BMI categories. CIMT was higher in diabetic than nondiabetic subjects (P < 0.0001) but showed no variation across BMI categories. Overall, CIMT correlated negatively with indices of antioxidant activity and positively with measures of lipid oxidation. Sex, age, BMI, and diabetes altogether explained 29.2% of CIMT, with no further improvement from adding PON1 and/or antioxidant status indices. Though indices of PON1 and oxidative status correlate with CIMT, their measurements may not be useful for identifying subjects at high CVD risk in this population.

## 1. Introduction

Early identification of people at high risk of atherosclerotic cardiovascular diseases (CVDs), followed by the implementation of lifestyle and drug interventions with proven beneficial effects, has been largely emphasized in strategies to reduce the mortality and morbidity from cardiovascular disease [1]. This is particularly relevant in some individuals including diabetic or obese people in whom risk factors for CVD tend to cluster and confer a very high risk of CVD [2]. Indeed, compared with their nondiabetic counterparts, people with type 2 diabetes have 2–6-fold higher risk for future CVD which accounts for up to 75% of mortality in this population [3]. The relation between adiposity and cardiovascular health was for a long time thought to be mediated solely by coincident CVD risk factors [4]. Several studies have however shown that obesity not only relates to but also independently predicts CVD [5, 6]. Nonetheless, there is no denying of the correlation between diabetes and obesity. Both conditions are increasingly viewed as proinflammatory states associated with an altered metabolic profile, endothelial dysfunction, and oxidative stress [5, 6]. Oxidative stress has been hypothesized as a mechanism linking the two conditions as well as accounting for their initiation, progression, and possible link with early atherosclerosis [5, 6]. It is plausible therefore that the association between increased body mass index (BMI) and diabetes and early atherosclerosis is largely due to underlying oxidative stress. The present study was undertaken to evaluate our hypothesis that specific indices of oxidative stress are associated with markers of subclinical CVD and may aid early CVD risk stratification in a population with a high prevalence of diabetes and obesity. For this purpose, indices of PON1 and oxidative status were determined by measuring levels and activities (paraoxonase and arylesterase) of paraoxonase (PON) 1, antioxidant activity (ferric reducing antioxidant power and trolox equivalent antioxidant capacity), and lipid peroxidation markers (malondialdehyde and oxidized LDL). The association of subclinical atherosclerosis with PON1 activity and oxidative stress has been reported in previous studies [7, 8]. Similar to the increasing rates of CVD worldwide, CVD contributes significantly to the public health burden in South Africa where interethnic differences in CHD prevalence and mortality are apparent [9]. The mixed ancestry is a heterogeneous South African ethnic group with a high risk for CVD demonstrated by very high prevalence of obesity, diabetes, and metabolic syndrome [10, 11]. Furthermore, we had also demonstrated high lifetime CVD risk in mixed-ancestry subjects with normoglycemia and those younger than 35 years [11].

## 2. Materials and Methods

### 2.1. Study Setting and Population

The study setting has been described in detail elsewhere [10, 11] and is located in a mixed-ancestry township (Bellville South) located within the Northern suburbs of Cape Town, Western Cape, South Africa. The mixed ancestry is a heterogeneous South African population comprising 32–43% Khoisan, 20–36% Bantu-speaking African, 21–28% European, and 9–11% Asian ancestry [12]. This specific study involved participants that took part in the survey during January 2011–November 2011. The study was approved by the research ethics committees of Stellenbosch University (Reference Number: N10/04/118) and the Cape Peninsula University of Technology (CPUT/HW-REC 2010/H017). The study was conducted according to the Code of Ethics of the World Medical Association (Declaration of Helsinki). All participants signed written informed consent after all the procedures were fully explained in the language of their choice.

### 2.2. Clinical Data

All consenting participants completed a questionnaire designed to obtain information on lifestyle factors such as smoking and alcohol consumption, physical activity, diet, family history of CVD, diabetes mellitus, and demographics. Blood pressure measurements were performed according to the World Health Organization (WHO) guidelines [13] using a semiautomatic digital blood pressure monitor (Rossmax, PA, USA) on the right arm in a sitting position. Other clinical measurements included the body weight, height, waist, and hip circumferences. Weight (to the nearest 0.1 kg) was determined in a subject wearing light clothing and without shoes and socks, using a Sunbeam EB710 digital bathroom scale, which was calibrated using a standard object of known mass. Waist circumference was measured using a nonelastic tape at the level of the narrowest part of the torso, as seen from the anterior view. The hip circumference was also measured using a nonelastic tape around the widest portion of the buttocks. All anthropometric measurements were performed three times and their average was used for analysis. Diabetes status was based on a history of doctor diagnosis, a fasting plasma glucose ≥7.0 mmol/L, and/or a 2-hour postoral glucose tolerance test (OGTT) plasma glucose >11.1 mmol/L as recommended by the WHO [14]. Adiposity was described according to body mass index (BMI in kg/m^2^) as normal weight (<25), overweight (25–30), and obese (>30).

### 2.3. Measurement of Carotid Intima-Media Thickness (CIMT)

Two qualified sonographers measured CIMT in longitudinal section at the far wall of the distal common carotid arteries, 2 cm from the bifurcation, at 3 consecutive end-points, 5–10 mm apart. The mean of six readings (3 from each side) was calculated for each participant using a portable B-mode and spectral Doppler ultrasound scanner equipped with cardiovascular imaging software. The GE LOGIQ e (General Electric Healthcare, Germany) high performance multipurpose colour compact ultrasound system included new imaging CrossXBeam technologies with multifrequency virtual apex on phased array cardiac transducer (3S-RS wide band phased probe 1.7–4.0 MHz) for echocardiography and a linear wideband vascular transducer (8L-RS 4.0–12 MHz linear probe) used for improved diagnostic confidence and imaging clarity for the carotids.

### 2.4. Laboratory Measurements

Blood samples were collected after an overnight fast and processed for further biochemical analyses. The Cobas 6000 immunometric analyzer (Roche Diagnostics) was used to measure levels of fasting plasma glucose (FBG), glycated haemoglobin (HbA1c), total cholesterol, high density lipoprotein cholesterol (HDL-c), triglycerides (TG) and *γ*-glutamyltransferase (GGT), and high sensitive C-reactive protein (hsCRP). Low density lipoprotein cholesterol (LDL-c) was calculated using Friedewald's formula [15]. An enzyme linked immunosorbent assay (ELISA) kit was used to measure plasma levels of paraoxonase 1 (PON1) following the manufacturer's instructions (Aviscera biosciences, Santa Clara, California). This assay employs a quantitative sandwich enzyme immunoassay technique.

### 2.5. Total Antioxidant Capacity

The total antioxidant capacity in plasma samples was assessed using the ferric reducing antioxidant power (FRAP) and trolox equivalent antioxidant capacity (TEAC) assays. FRAP was done according to the method of Benzie and Strain [16]. Briefly, plasma samples were mixed with FRAP reagent, incubated for 30 min at 37°C, and the absorbance at 593 nm was recorded using a spectrophotometer (Spectramax plus384 Molecular devices, USA). The TEAC assay was according to Re et al. [17] and is based on monitoring (at 734 nm) the oxidation of 2, 2′-azinobis-(3-ethylbenzothiazoline-6-sulfonic acid) radical (ABTS) cation formed by reacting ABTS, and potassium persulfate. Distilled water was used instead of PBS to dilute the ABTS^+^ radical solution.

### 2.6. Paraoxonase Activity

Paraoxonase (PONase) and arylesterase (AREase) activities were measured using paraoxon and phenylacetate (Sigma Aldrich, SA) as substrates, respectively. PONase activity was measured using Richter and Furlong's method [18] from the initial velocity of p-nitrophenol production at 37°C and the increased absorbance at 405 nm was monitored on a spectrophotometer (Spectramax plus384, Molecular devices, USA). Each serum sample was incubated with 5-mmol/L eserine (Sigma Aldrich, SA) for 15 minutes at room temperature to inhibit serum cholinesterase activity which is usually elevated in diabetes and would otherwise interfere with the determination of paraoxonase activity in serum from diabetic individuals. PON-1 activity of 1 U/L was defined as 1 *μ*mol of p-nitrophenol hydrolyzed per minute. A slightly modified method of Browne et al. [19] was used to measure AREase activity. The working reagent consisted of 20 mmol/L Tris-HCl, 4 mmol/L phenyl acetate, pH 8.0, with 1.0 mmol/L CaCl_2_ (Sigma Aldrich, SA). The reaction was initiated by adding 5 *μ*L of 40-fold tris-diluted samples to 345 *μ*L of the working reagent at 25°C. The change in absorbance at 270 nm was recorded for 60 minutes after a 20-second lag time on a Spectramax plus384 spectrophotometer. The activity, expressed as kU/L, was based on the molar absorptivity (1310) of phenol at 270 nm. In both assays, the rates used to generate the data points were derived from the linear portions of the rate versus time plots.

### 2.7. Lipid Peroxidation

Plasma MDA and ox-LDL were used as markers of lipid peroxidation (LPO). The method of Jentzsch et al. [20] was used to estimate the thiobarbituric acid reactive substances (TBARS) which reflect the production of MDA. Plasma ox-LDLs were measured using a quantitative sandwich ELISA kit (Cellbiolabs, San Diego, California).

### 2.8. Statistical Analyses

Data are presented as mean standard deviation, SD, or median of 25th–75th percentiles for continuous variables and as count and percentage for categorical variables. For group (sex, diabetes status, and BMI, quarters of CIMT) comparisons, chi square test, student's *t*-test, and analysis of the variance (ANOVA) and nonparametric equivalents were used. Continuous associations between CIMT and the indices were assessed graphically with the use of correlation matrix, before and after applying the Box-Cox [21] power transformations to improve the shape of the associations; then the “Covariance Estimation for Multivariate *t* Distribution” [22] method was used to derive the correlation coefficients, while minimizing the potential effects of outliers. The Steiger *t*-test was used to compare correlation coefficients among indices. Regression coefficients to indicate the size of the association of each of the indices with CIMT were derived from robust multiple linear regression models that included each of the 4 variables of interest, age, sex, body mass index, and diabetes status. Analyses were carried out using R statistical software version 3.0.0 [03-04-2013], (The R Foundation for Statistical Computing, Vienna, Austria). The significance level was set at 0.05.

## 3. Results

### 3.1. Participants' Basic Profile

Of the 651 participants (men 170, 26%) who took part in the study, 160 (25%) were excluded from this analysis as they had missing values for CIMT and/or other relevant variables. Baseline characteristics of participants included and excluded from analyses were very similar. The final analytic sample comprised 491 participants (men 126, 25.7%) with a mean age of 54.6 (13.2) years. Among them, 142 (29%) had diabetes, 137 (28%) were overweight, and 261 (53%) were obese. The average BMI was 31.4 (8.1) kg/m^2^ (Table 1). There were no age differences between men and women and across the BMI profiles but diabetic subjects were significantly older than nondiabetic ones (59.6 versus 52.5 years, *P* < 0.0001) and had higher BMI (33.4 versus 30.6 kg/m^2^, *P* = 0.002). Women had significantly higher levels of HbA1c, BMI, and waist circumference. In general, there were no differences between the genders with regard to the lipid profile. Triglyceride levels increased while HDL-cholesterol decreased across BMI categories (both *P* < 0.0001, ANOVA).

### 3.2. Paraoxonase and Oxidative Status Profile

Men had significantly higher FRAP (732 versus 655 *μ*M, *P* = 0.006) and ox-LDL (5141 versus 4110 ng/mL, *P* < 0.0001) and lower AREase activity and PON 1 levels (91 versus 117 kU/L; 88 versus 98 *μ*g/mL, *P* < 0.0001) respectively, compared to women. In diabetic subjects, a less favorable profile was observed for PON1 (mass and activity) and oxidative status (decreased FRAP and TEAC; elevated Ox-LDL and TBARS). A similar less favorable profile was also apparent across increasing BMI categories (Table 1).

### 3.3. CIMT Profile and Associations with PON1 and Oxidative Profiles

**Figure 1:**
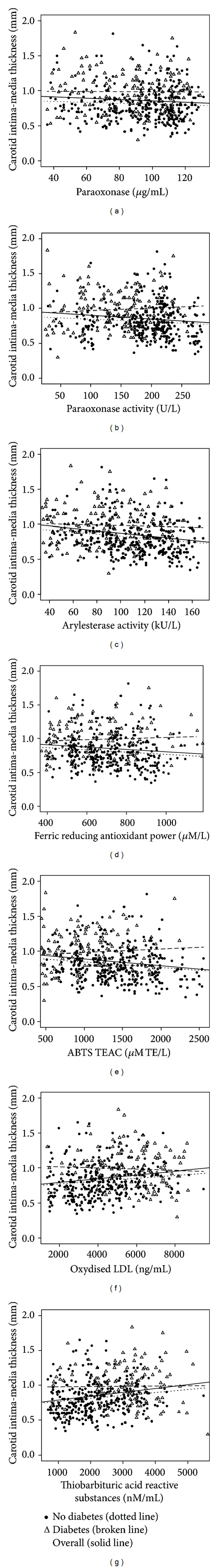
Correlations of paraoxonase, paraoxonase activity, arylesterase ferric reducing antioxidant power, TEAC, oxidized LDL, and thiobarbituric acid reactive substances activity with carotid intima-media thickness. Participants are grouped by diabetes status with the triangles representing those with diabetes and the solid circles representing those without diabetes. The superimposed regression lines are for the overall sample (solid line), and participants with (broken line) and without diabetes (dotted lines). Accompanying correlations coefficients are shown in Table 2.

The median CIMT was 0.82 mm. It was higher in men than in women (0.95 versus 0.80 mm, *P* < 0.0001) and in diabetic than in nondiabetic subjects (0.98 versus 0.77 mm, *P* < 0.0001). However, there was neither a significant difference (*P* > 0.227) nor a linear trend in the distribution of CIMT levels across BMI categories (Table 1). Overall, CIMT correlated negatively with all indices of antioxidant activity and positively with the measures of lipid oxidation (Table 2, Figure 1). Correlation coefficients however were very weak, with borderline significant differences by diabetes status for the correlations of CIMT with TEAC (*P* = 0.04), Ox-LDL (*P* = 0.02), and TBARS (*P* = 0.04). In stratified analyses, the correlation coefficients for each of these three indices always appeared to be significant and stronger in nondiabetics and weak and nonsignificant in diabetics (Table 2, Figure 1). The distribution of participants' characteristics across quarters of CIMT is shown in Table 3 showing increasing age, systolic blood pressure, waist/hip ratio, fasting glucose, total cholesterol, and decreasing proportion of women across increasing quarters of CIMT.

### 3.4. Multivariable Analysis

In a model comprising sex, age, and BMI, each of the three variables was significantly associated with CIMT. This basic model explained 26.4% of the variation in CIMT levels. When this model was expanded with the inclusion of diabetes status, all variables in the model remained significantly associated with CIMT and further explained 29.2% of the variation. However, as shown in Table 4, none of the indices of PON1 and antioxidant status significantly associated with CIMT in the presence of the four basic variables and furthermore did not improve the fit of models in predicting the variability of CIMT. In the presence of variables in the basic model, SBP (*β* = 0.002, *P* < 0.0001), but not DBP (*β* = 0.001, *P* = 0.066), was significantly associated with CIMT. In the same basic model, neither waist circumference (*β* = −0.0007, *P* = 0.434) nor waist/hip ratio (*β* = 0.031, *P* = 0.724) was associated with CIMT, even when they were allowed to replace BMI in the model (both *P* > = 0.364).

## 4. Discussion

The prominent role of oxidative stress in the occurrence of atherosclerosis and related complications is well recognized, with suggestions that measurement of oxidative stress may aid risk stratification and effective prevention of atherothrombotic diseases. In this study, we evaluated the profile of several indices of PON1 and oxidative status and their relationship with CIMT, which is a marker of subclinical cardiovascular disease. Our findings demonstrate that measures of oxidative stress together with PON1 are only superficially associated with CIMT. Instead, conventional risk factors such as age, gender, adiposity, and chronic hyperglycemia and not measures of oxidative stress may be more important in estimating CVD risk in our population.

The physiological role of PON1 in reducing atherosclerosis stems from its ability to inhibit the oxidation of low density lipoprotein (LDL) [23] and its ability to stimulate cholesterol efflux from macrophages [24]. In this regard, decreased activities of PON1 and/or other antioxidant species have been demonstrated in obesity, diabetes, and other oxidative stress-related conditions [25–27]. In our study, we found PON1 activities to be significantly reduced in obese and diabetic subjects. It has previously been suggested that decreased PON1 activity in diabetes may be due to glycation-induced changes to HDL and/or PON1, thereby affecting its association with HDL that has been related to its antiatherogenic properties [28]. Similar to diabetes, obesity is strongly associated with oxidative stress and proinflammatory state which in this study is corroborated by significantly raised oxidative stress markers (ox-LDL and TBARS) in obese subjects.

Proinflammatory markers and oxidative stress have been shown to modulate and inactivate PON1 activity [29–32]. Adipose tissue expresses inflammatory cytokines, interleukin-6 (IL-6), and tumor necrosis factor-*α* (TNF-*α*) which are associated with oxidative stress [33]. In a study which introduced a mixture of IL-6, IL-1, and TNF-*α* in murine hepatoma cell line Hepa 1–6, a reduction in PON1 mRNA was observed [29]. In addition, obesity alters the composition of HDL in a manner that may impair binding of PON1 to HDL surface such as lowering both HDL's largest subfraction (HDL2) and its major binding protein (apo A1) [34]. Since PON1 is a lipid-dependent enzyme whose activity hinges on its conformation within HDL, the impaired binding in results decreased enzyme activity.

Measurements of oxidative stress have previously been proposed as a predictor of atherosclerosis in end stage renal disease patients [7]. In their study, Dursan et al. [7] demonstrated significant positive correlation between CIMT and serum TBARS and nitrite/nitrate levels and a significant negative correlation between CIMT and antioxidant markers superoxide dismutase (SOD), catalase (CAT), and plasma sulfhydryl (P-SH) levels in patients on chronic haemodialysis. We found total antioxidants (FRAP, AREase) to be negatively correlated with CIMT, whilst markers of oxidative stress (ox-LDL and TBARS) showed a positive correlation, but the association was not retained in further adjusted regression analyses and there were suggestions that diabetes affects these associations since they were generally stronger and significant in nondiabetics compared to diabetics. Instead, traditional CVD risk factors, age, gender, obesity, and diabetes, were significant determinants of subclinical atherosclerosis, accounting for 29.2% of CIMT variability. Previous studies have demonstrated that only a fraction of CVD risk is explained by traditional risk factors [35, 36] prompting a search for alternate and additional predictors. Emerging data from around the world support the pivotal role of chronic inflammation in the occurrence of CVD complications. Although influences of PON1 and oxidative stress have been demonstrated to be on the early steps of atherosclerosis [37], our results exclude measurements of PON1 activity and indices of antioxidant status in prediction of atherosclerotic risk.

Some limitations should be accounted for when interpreting our findings. First, the cross-sectional design of our study precludes drawing inferences on the direction of the associations. Second, we did not establish the intraobserver variability between the sonographers who performed CIMT measurements; however we used multiple measurements at different points. Third, because our study population was ethnically exclusive, our results can only be generalized to mixed-ancestry South African subjects. Fourth, we used BMI as the marker of obesity although it cannot distinguish between visceral and subcutaneous fat. Visceral fat is particularly strongly associated with atherosclerotic CVD risk [38]. Fifth, we did not evaluate other residual confounders that may affect oxidative stress markers such as dietary factors. Lastly, the number of males versus that of females is skewed, with this being a common trend in South African population studies. Nonetheless, we used two methods for each of the three measures of oxidative status. Besides allowing us to demonstrate the consistency of our findings, this approach reinforces the accuracy of antioxidant status results since the measured total antioxidant status of biological samples is known to be method specific [39]. Furthermore, the nonspecific nature of the MDA-TBARS method requires corroboration. Since PON1 is purported to function as an antioxidant, another key strength distinguishing this study from several others is the evaluation of its activity in the context of antioxidant status.

In conclusion, although atherosclerosis is considered an inflammatory/oxidative condition, our results argue against a major role of PON1 and oxidative status in prediction of atherosclerotic risk as none of these indices impacted on the model's value in explaining the variability of CIMT. Rather, the findings reaffirm the importance of conventional risk factors such as age, gender, adiposity, and chronic hyperglycemia in estimating CVD risk in this mixed-ancestry population.

## Figures and Tables

**Table 1:** General characteristics of the participants.

Variables	Overall	Sex			Diabetes			BMI			
		Men	Women	*P*	No	Yes	*P*	Normal	Overweight	Obese	*P*
*N*	491	126	365		349	142		93	137	261	
Female, *n* (%)	365 (74.3)	0	365 (100)		257 (73.6)	108 (76.1)	0.578	47 (50.5)	93 (67.9)	235 (90.0)	<0.0001
Age (years)	54.6 (13.2)	55.4 (13.3)	54.3 (13.2)	0.401	52.5 (13.4)	59.6 (11.5)	<0.0001	55.3 (15.2)	53.6 (13.3)	54.8 (12.6)	0.568
BMI (kg/m^2^)	31.4 (8.1)	27.5 (7.0)	32.8 (8.0)	<0.0001	30.6 (7.3)	33.4 (9.5)	0.002	21.5 (3.1)	27.6 (1.4)	37.0 (6.7)	<0.0001
Waist circumference (cm)	96.4 (15.4)	94.9 (14.5)	96.9 (15.7)	0.208	94.6 (16.1)	100.8 (12.9)	<0.0001	80.5 (17.1)	90.7 (8.4)	105.1 (11.2)	<0.0001
Waist/hip ratio	0.89 (0.12)	0.93 (0.12)	0.87 (0.12)	<0.0001	0.88 (0.12)	0.92 (0.12)	0.001	0.85 (0.19)	0.89 (0.09)	0.90 (0.10)	0.010
Systolic BP (mmHg)	136 (26)	138 (24)	136 (27)	0.309	133 (23)	144 (31)	0.0001	133 (31)	135 (25)	136 (25)	0.138
Diastolic BP (mmHg)	82 (14)	84 (15)	81 (13)	0.030	81 (14)	84 (14)	0.055	78 (12)	81 (12)	83 (15)	0.002
FPG (mmol/L)	6.4 (2.9)	6.2 (2.5)	6.5 (3.1)	0.292	5.1 (0.7)	9.5 (3.9)	<0.0001	5.3 (1.5)	6.6 (3.4)	6.7 (3.0)	0.0003
HbA1c (%)	6.6 (1.6)	6.3 (1.4)	6.7 (1.7)	0.025	5.9 (0.4)	8.3 (2.2)	<0.0001	5.9 (1.0)	6.8 (2.0)	6.7 (1.6)	<0.0001
Creatinine (µM)	74.8 (25.5)	89.5 (35.5)	69.6 (18.5)	<0.0001	75.3 (20.0)	81.1 (42.8)	0.21	79.4 (19.1)	75.0 (19.3)	73.7 (30.0)	0.480
C-reactive protein (mg/L)	5.1 [2.0–8.9]	3.7 [1.5–7.6]	5.6 [2.2–9.2]	0.025	5.0 [1.9–8.4]	5.5 [2.5–10.2]	0.126	3.0 [1.0–5.6]	2.9 [1.4–6.6]	6.8 [3.5–11.1]	0.375
GGT (IU/L)	27 [19–43]	31 [24–46]	25 [17–41]	<0.0001	25 [18–41]	29 [23–50]	0.003	26 [18–42]	25 [18–43]	28 [20–43]	0.451
Total cholesterol (mmol/L)	5.5 (1.1)	5.4 (1.0)	5.5 (1.1)	0.494	5.5 (1.1)	5.5 (1.2)	0.360	5.4 (1.1)	5.7 (1.1)	5.4 (1.0)	0.072
HDL cholesterol (mmol/L)	1.4 (0.4)	1.3 (0.5)	1.4 (0.4)	0.058	1.4 (0.4)	1.3 (0.4)	0.009	1.6 (0.5)	1.3 (0.4)	1.3 (0.3)	<0.0001
LDL cholesterol (mmol/L)	3.4 (1.0)	3.4 (0.9)	3.4 (1.0)	0.525	3.4 (0.9)	3.4 (1.0)	0.990	3.3 (1.1)	3.6 (1.0)	3.4 0.9)	0.026
Triglycerides (mmol/L)	1.5 (0.9)	1.7 (1.1)	1.5 (0.8)	0.047	1.4 (0.8)	1.8 (1.0)	<0.0001	1.2 (0.8)	1.6 (0.9)	1.6 (0.9)	0.0001
CIMT (mm)	0.82 [0.67–1.02]	0.95 [0.72–1.22]	0.80 [0.65–0.95]	<0.0001	0.77 [0.65–0.93]	0.98 [0.77–1.15]	<0.0001	0.85 [0.65–1.08]	0.80 [0.70–0.92]	0.82 [0.68–1.00]	0.227
PON1 (µg/mL)	96 [74–111]	88 [69–105]	98 [78–112]	0.002	99 [77–112]	91 [66–107]	0.0005	107 [92–115]	99 [77–111]	91 [71–108]	0.0006
PONase (U/L)	184 [131–220]	194 [158–218]	183 [124–221]	0.091	204 [167–225]	156 [96–182]	<0.0001	221 [186–244]	198 [156–226]	172 [102–206]	<0.0001
AREase (kU/L)	107 [82–132]	91 [65–104]	117 [90–137]	<0.0001	113 [91–137]	90 [63–115]	<0.0001	118 [96–147]	107 [80–132]	98 [77–123]	<0.0001
FRAP (µM)	677 [545–827]	732 [592–861]	655 [537–811]	0.006	722 [588–852]	614 [471–732]	<0.0001	754 [642–911]	725 [583–852]	638 [523–749]	<0.0001
TEAC (nM)	1279 [944–1654]	1366 [1018–1742]	1271 [936–1631]	0.086	1423 [1077–1746]	1051 [681–1320]	<0.0001	1570 [1238–1882]	1366 [1153–1846]	1206 [879–1505]	<0.0001
Ox-LDL (ng/mL)	4408 [3143–5911]	5141 [3979–6333]	4110 [2834–5484]	<0.0001	3916 [2755–5138]	5885 [4602–6944]	<0.0001	3535 [2511–4747]	4337 [2859–5736]	4780 [3683–6216]	<0.0001
TBARS (nM)	2277 [1559–3132]	2042 [1527–2772]	2304 [1625–3214]	0.203	1982 [1406–2672]	3152 [2380–3655]	<0.0001	1546 [1046–2369]	2238 [1546–3115]	2533 [1924–3281]	<0.0001

FPG: fasting plasma glucose; BMI: body mass index; BP blood pressure; GGT: gamma glutamyl transferase; HDL: high density lipoprotein; LDL: low density lipoprotein; CIMT: carotid intima-media thickness; PON1: paraoxonase 1; PONase: paraoxonase; AREase: arylesterase; FRAP: ferric reducing ability of plasma; TEAC: trolox equivalent antioxidant capacity; Ox-LDL: oxidized LDL; TBARS: thiobarbituric acid reactive substances.

**Table 2:** Robust correlation of CIMT with PON and antioxidant status.

Correlates	Overall	No diabetes	Diabetes	*P* value
PON1	−0.09 (−0.17 to 0.00)	−0.04 (−0.14 to 0.07)	−0.01 (−0.17 to 0.16)	0.74
PONase	−0.17 (−0.25 to −0.08)	−0.11 (−0.21 to 0)	0.04 (−0.13 to 0.20)	0.15
AREase	−0.24 (−0.32 to −0.15)	−0.20 (−0.30 to −0.09)	−0.05 (−0.21 to 0.12)	0.13
FRAP	−0.17 (−0.26 to −0.09)	−0.12 (−0.22 to −0.02)	0.03 (−0.13 to 0.20)	0.12
TEAC	−0.22 (−0.30 to −0.13)	−0.16 (−0.26 to −0.06)	0.04 (−0.12 to 0.21)	0.04
Ox-LDL	0.25 (0.17 to 0.34)	0.21 (0.10 to 0.30)	−0.03 (−0.20 to 0.13)	0.02
TBARS	0.26 (0.17 to 0.34)	0.19 (0.09 to 0.29)	−0.01 (−0.17 to 0.16)	0.04

Paraoxonase 1; PONase: paraoxonase; AREase: arylesterase; FRAP: ferric reducing ability of plasma; TEAC: trolox equivalent antioxidant capacity; Ox-LDL: oxidized LDL; TBARS: thiobarbituric acid reactive substances.

**Table 3:** Characteristics of the participants by quarters of CIMT.

Variables	Quartiles of CIMT				*P* value
	Q1	Q2	Q3	Q4	
N	123	131	118	119	NA
CIMT [min–max]	0.30–0.67	0.68–0.82	0.83–1.02	1.03–2.18	NA
Female, n (%)	101 (82.1)	104 (79.4)	97 (82.2)	63 (52.9)	<0.0001
Age (years)	44.9 (12.9)	53.3 (11.8)	57.6 (11.3)	63.0 (9.8)	<0.0001
BMI (kg/m^2^)	30.5 (6.7)	31.5 (8.0)	32.3 (8.0)	31.5 (9.5)	0.234
Waist circumference (cm)	94.0 (18.7)	97.1 (13.5)	96.3 (14.0)	98.2 (14.9)	0.060
Waist/hip ratio	0.86 (0.15)	0.87 (0.12)	0.89 (0.10)	0.93 (0.10)	<0.0001
Systolic BP (mmHg)	125 (19)	135 (26)	138 (21)	149 (31)	<0.0001
Diastolic BP (mmHg)	78 (12)	83 (16)	82 (11)	84 (15)	0.007
FPG (mmol/L)	5.7 (2.2)	6.0 (2.3)	6.4 (2.8)	7.6 (3.9)	<0.0001
HbA1c (%)	6.2 (1.1)	6.2 (1.0)	6.7 (1.6)	7.4 (2.3)	<0.0001
Creatinine (µM)	68.8 (14.3)	72.8 (19.0)	72.2 (18.6)	85.8 (39.8)	<0.0001
C-reactive protein (mg/L)	4.4 [1.4–7.6]	5.1 [2.2–8.7]	5.4 [2.5–9.2]	5.3 [2.0–9.2]	0.222
GGT (IU/L)	24 [17–38]	29 [19–49]	28 [19–41]	29 [21–43]	0.145
Total cholesterol (mmol/L)	5.2 (0.9)	5.4 (1.1)	5.7 (1.2)	5.7 (1.1 )	<0.0001
HDL cholesterol (mmol/L)	1.4 (0.4)	1.3 (0.4)	1.4 (0.4)	1.4 (0.4)	0.708
LDL cholesterol (mmol/L)	3.2 (0.8)	3.4 (1.0)	3.6 (1.0)	3.6 (1.0)	0.003
Triglycerides (mmol/L)	1.3 (0.7)	1.5 (0.9)	1.7 (1.0)	1.6 (0.9)	0.0008
PON1 (µg/mL)	102 [78–112]	94 [77–110]	98 [77–112]	94 [70–110]	0.290
PONase (U/L)	204 [170–233]	184 [130–215]	182 [136–218]	177 [119–217]	0.005
AREase (kU/L)	118 [97–141]	103 [84–126]	94 [75–132]	95 [76–120]	<0.0001
FRAP (µM)	734 [602–887]	672 [573–809]	636 [533–795]	667 [534–785]	0.014
TEAC (nM)	1484 [1068–1858]	1316 [947–1628]	1206 [929–1595]	1235 [904–1568]	0.001
Ox-LDL (ng/mL)	3618 [2641–5102]	4576 [3230–5844]	4747 [3638–6176]	4975 [3545–6333]	<0.0001
TBARS (nM)	1857 [1251–2504]	2280 [1544–3042]	2447 [1919–3256]	2571 [1905–3666]	<0.0001

FPG: fasting plasma glucose; BMI: body mass index; BP blood pressure; GGT: gamma glutamyl transferase; HDL: high density lipoprotein; LDL: low density lipoprotein; CIMT: carotid intima-media thickness; PON1: paraoxonase 1; PONase: paraoxonase; AREase: arylesterase; FRAP: ferric reducing ability of plasma; TEAC: trolox equivalent antioxidant capacity; Ox-LDL: oxidized LDL; TBARS: thiobarbituric acid reactive substances.

**Table 4:** Regression coefficients from multiple robust linear models for the prediction of CIMT by indices of PON1 and antioxidant status accounting for the potential effect of sex, age, diabetes, and adiposity.

	Age—sex—BMI		Diabetes		PON1		PONase		AREase		FRAP		TEAC		Ox-LDL		TBARS	
	*β*	*P*	*β*	*P*	*β*	*P*	*β*	*P*	*β*	*P*	*β*	*P*	*β*	*P*	*β*	*P*	*β*	*P*
**Index**	—	—	—	—	−0.0002	0.723	−0.00006	0.730	−0.0003	0.390	−0.00002	0.743	−0.00002	0.571	0.000004	0.539	0.00001	0.255
Age	0.009	<0.0001	0.008	<0.0001	0.008	<0.0001	0.008	<0.0001	0.008	<0. 0001	0.008	<0.0001	0.008	<0.0001	0.008	<0.0001	0.008	<0.0001
Sex (men)	0.151	<0.0001	0.150	<0.0001	0.149	<0.0001	0.150	<0.0001	0.141	<0.0001	0.151	<0.0001	0.150	<0.0001	0.145	<0.0001	0.145	<0.0001
BMI	0.004	0.002	0.003	0.017	0.003	0.029	0.003	0.030	0.003	0.041	0.003	0.030	0.003	0.035	0.003	0.027	0.003	0.043
Diabetes	—	—	0.124	<0.0001	0.122	<0.0001	0.121	<0.001	0.118	<0.0001	0.121	<0.0001	0.120	<0.0001	0.119	<0.0001	0.114	<0.0001
*R* ^2^	0.264		0.292		0.292		0.293		0.291		0.295		0.293		0.292		0.294	

BMI: body mass index; paraoxonase 1; PONase: paraoxonase; AREase: arylesterase; FRAP: ferric reducing ability of plasma; TEAC: trolox equivalent antioxidant capacity; Ox-LDL: oxidized LDL; TBARS: thiobarbituric acid reactive substances.
